# BRAF immunohistochemistry predicts sentinel lymph node involvement in intermediate thickness melanomas

**DOI:** 10.1371/journal.pone.0216043

**Published:** 2019-04-30

**Authors:** Atte A. Manninen, Maria Gardberg, Susanna Juteau, Suvi Ilmonen, Joonas Jukonen, Noora Andersson, Olli Carpén

**Affiliations:** 1 Department of Plastic Surgery, University of Helsinki and Helsinki University Hospital, Helsinki, Finland; 2 Institute of Biomedicine, Research Center for Cancer, Infections and Immunity, University of Turku and Turku University Hospital, Turku, Finland; 3 Department of Pathology, University of Helsinki and Helsinki University Hospital, Helsinki, Finland; 4 Institute of Biomedicine, University of Helsinki, Helsinki, Finland; 5 Research Program in Systems Oncology, University of Helsinki, Helsinki, Finland; University of Queensland Diamantina Institute, AUSTRALIA

## Abstract

**Background:**

Sentinel node biopsy (SNB) is an important step in melanoma staging and prognostication. It is commonly performed for patients with intermediate thickness melanomas, based on clinicopathological features. However, only 20–25% of patients eventually demonstrate nodal involvement. The aim of this study was to evaluate whether tissue biomarkers with links to melanoma biology, together with clinicopathological parameters, could aid in the prediction of sentinel node involvement and improve selection of patients for SNB. In addition, we examined the role of these clinical or biological markers in disease outcome.

**Methods:**

We collected a case-control cohort of 140 intermediate thickness (Breslow 0,9–4,0mm) melanoma patients with or without SNB involvement matched for age, gender, Breslow thickness and location. From this cohort, we tested the predictive value of common clinicopathological parameters (ulceration, mitotic count and tumor regression) and FMNL-2, ezrin and BRAF V600E immunoreactivity, for sentinel node involvement and survival. We further analyzed the correlations in the superficial spreading melanoma subtype.

**Results:**

Based on our case control analysis, of the markers, BRAF V600E status (p = 0.010) and mitotic count (p = 0.036) correlated with SNB involvement. SNB status was a strong independent prognosticator for recurrence free survival (RFS p<0.001), melanoma specific survival (MSS p = 0.000) and overall survival (OS p = 0.029). In the superficially spreading melanoma subgroup, BRAF V600E positivity indicated poorer RFS (p = 0.039) and OS (p = 0.012). By combining the Breslow thickness, mitotic count and BRAF immunohistochemistry, we identified a group of superficially spreading melanomas with an excellent survival probability independent of SNB status.

**Conclusions:**

These results demonstrate that BRAF immunohistochemistry could serve as a useful addition to a marker panel for selecting intermediate thickness melanoma patients for SNB.

## Introduction

Cutaneous melanoma is a common malignant neoplasia with over 230 000 cases and 55 000 cancer deaths annually [1]. While death rates are projected to remain stable, melanoma incidence and treatment costs are estimated to rise significantly through 2030 [2].

The dissemination and prognosis of melanoma is evaluated by the TNM-classification at the time of diagnosis, assessing thickness (Breslow) and ulceration of the primary tumor (T), lymph node involvement (N) and presence of distant metastases (M) [3]. According to TNM-classification, tumors are categorized into four stages, where stage I and II are local, stage III includes positive regional lymph nodes and stage IV has distant metastases. Melanoma prognosis declines as the stage increases and in stage IV, the 5-year survival is only 15–20% [4]. At present, sentinel node biopsy (SNB) has proven to be the most important prognostic factor for melanoma specific survival and overall survival in patients with cutaneous melanoma thicker than 1mm [5]. In several recommendations, SNB is performed for melanomas thicker than 0.9 mm. If the tumor is ulcerated, SNB may be performed even in thinner melanomas. Even though uncommon, these superficial melanomas occasionally metastasize [6]. Standard of care in the intermediate thickness (1.0–4.0 mm Breslow) melanoma includes radical excision of the primary tumor and concomitant SNB. However, in this patient group, only 20–25% of patients have affected sentinel lymph nodes. This means that up to five operations are needed to detect a single lymph node positive patient [7]. There is a clear need for markers that would improve the preoperative identification of sentinel lymph node positive/negative patients for targeting individuals for SNB. Currently, such biomarkers have not been described.

In melanoma, the commonly activated oncogenic signaling pathways include MAPK (mitogen activated protein kinase) and the PI3K (phosphoinositide-3-OH kinase). Over-activation of these pathways leads to increased cell proliferation and cancer cell survival [8]. Abnormal activation is typically induced by oncogenic mutations. In melanomas, the most commonly mutated oncogene is BRAF present in more than 50% of tumors [9]. Ninety percent of all activating BRAF mutations involve V600E substitution [10]. BRAF V600E increases the invasive potential of melanoma cells, but the down-stream effectors are not well known [11].

Formins constitute a protein family with diverse actin-regulating and potentially pro-invasive functions linked to formation of protrusions of lamellipodia at the cell migrating edge [12]. The family includes two homologous members, formin-like protein 2 (FMNL2) and formin-like protein 3 (FMNL3). Previous studies have suggested that especially FMNL2 participates in melanoma cell invasion by driving elongation of actin filaments that constitute the lamellipodia [13,14]. Gardberg et al. further analyzed the role of FMNL2 in invasive properties of melanoma and melanoma outcome. In stage I-II melanoma patients, FMNL2 expression was an independent predictor of survival together with melanoma thickness [15]. Interestingly, in BRAF V600E mutant cells, the specific inhibitor vemurafenib, reduces FMNL2 expression [15]. Another actin-modulating protein involved in cell motility is ezrin, an adaptor protein that links the cytoskeleton to the plasma membrane [16]. Ezrin can promote tumor invasion [17], and in various malignancies, including uveal melanoma, is significantly associated with outcome of the disease [18]. Ezrin expression is significantly higher in cutaneous melanomas than in benign naevi and slightly higher expression levels are observed in metastatic than in primary melanomas [19].

In the present study, our aim was to study potential predictors of sentinel node involvement in a cohort of patients with intermediate thickness melanoma. Apart from the common clinicopathological markers, we focused on BRAF V600E mutation detected by immunohistochemistry and the immunoreactivity of FMNL2 and ezrin. In addition, we analyzed the value of these markers in predicting disease outcome.

## Methods

### Patient cohort

The cohort consisted of patients with primary cutaneous melanoma operated at the Helsinki University Hospital, Helsinki, Finland in 2007–2015, altogether 770 patients. Patient records were reviewed, and 100 consecutive sentinel node biopsy—positive (SNB+) patients who met the inclusion criteria were identified. The inclusion criteria were: positive sentinel node biopsy, age during surgery (18–80 years), and intermediate thickness melanoma (Breslow 0.9–4.0mm). Eighteen patients were excluded as the primary melanoma sample was missing. We then selected a matching primary melanoma control group of patients whose sentinel node was negative (SNB-). The case—control matching criteria were: patient age (+/- 5 years), same anatomical location of the primary tumor (head and neck, trunk, upper limb or lower limb), same sex and same melanoma thickness (+/- 0.2 mm). Patients whose primary melanoma sample could not be recovered, were excluded from the study. This resulted in a final study cohort of 70 SNB+ patients and 70 matching SNB-negative controls (N = 140).

All patients underwent wide local excision with histologically clear margins and SNB. If sentinel node was found positive, the patients then underwent a secondary surgery for the nodal basin, where the positive sentinel node was located (axillary clearance, groin evacuation or neck dissection) according to clinical guidelines. Additional treatment included chemotherapy for eighteen, radiotherapy for twenty-five and interferon for ten patients. In four cases, the isolated limb perfusion was also used. The follow up was performed at the Department of Plastic Surgery or at the Department of Oncology at Helsinki University Hospital. The final follow-up date was defined as the date of the last follow-up visit or the date of death. Most recent follow-up information was gathered from the medical records in August 2017. Median follow up time was 5.1 years (range 2 months– 10 years). The cause of death was obtained from the patient records and autopsy reports.

The Ethical Committee of Surgery of the University of Helsinki approved the use of the collected tissue samples and associated clinical information for this study. Ethical committee waived the need for informed consent from patients since no new specimen was collected.

### Primary melanoma samples and SNB–specimens

New sections were cut from paraffin embedded specimens and stained with H&E. Relevant pathological parameters (Breslow thickness, ulceration, mitotic count, tumor type, regression) were evaluated by an experienced dermatopathologist. If the parameters differed from those reported in the primary pathological report they were corrected. Sentinel nodes were sliced at 1 mm intervals and analyzed by routine H&E-staining and immunohistochemistry (IHC) (S-100, HMB-45 and MART-1). From the sentinel node, S-100, MART-1 and HMB-45 IHC was performed to identify metastatic cells. The size and location and possible multifocality of the metastasis were examined and reported. Isolated tumor cells (ITC) were also deemed positive as a metastasis.

For BRAF V600E analysis, 3 um thick melanoma sections were stained with Ventana Benchmark Ultra (Roche/Ventana, Tucson, AZ, USA). The protocol was based on heat-induced epitope retrieval using standard pretreatment buffer CC1, 64 min/98°C. The slides were incubated with primary antibody, BRAF V600E that specifically recognizes the BRAF V600E-mutation, (clone VE1, RTU, 790–4855, Roche/Ventana) for 44 min/36°C. Antigen detection was performed with biotin-free, three step multimer- based detection kit Optiview (760–700, Roche/Ventana, Tucson, AZ, USA). The slides were dehydrated and mounted. Hematoxylin staining was performed with slide staining instrument SAKURA TISSUE-TEK PRISMA (Sakura Finetek Europe B.V., The Netherlands). For detailed description of the method, see [20]. The stained specimens were then examined by two investigators (AM and SJ) and labeled as positive or negative. The evaluators were blinded for the follow-up data, sentinel node positivity and immunohistochemical results.

**Fig 1:**
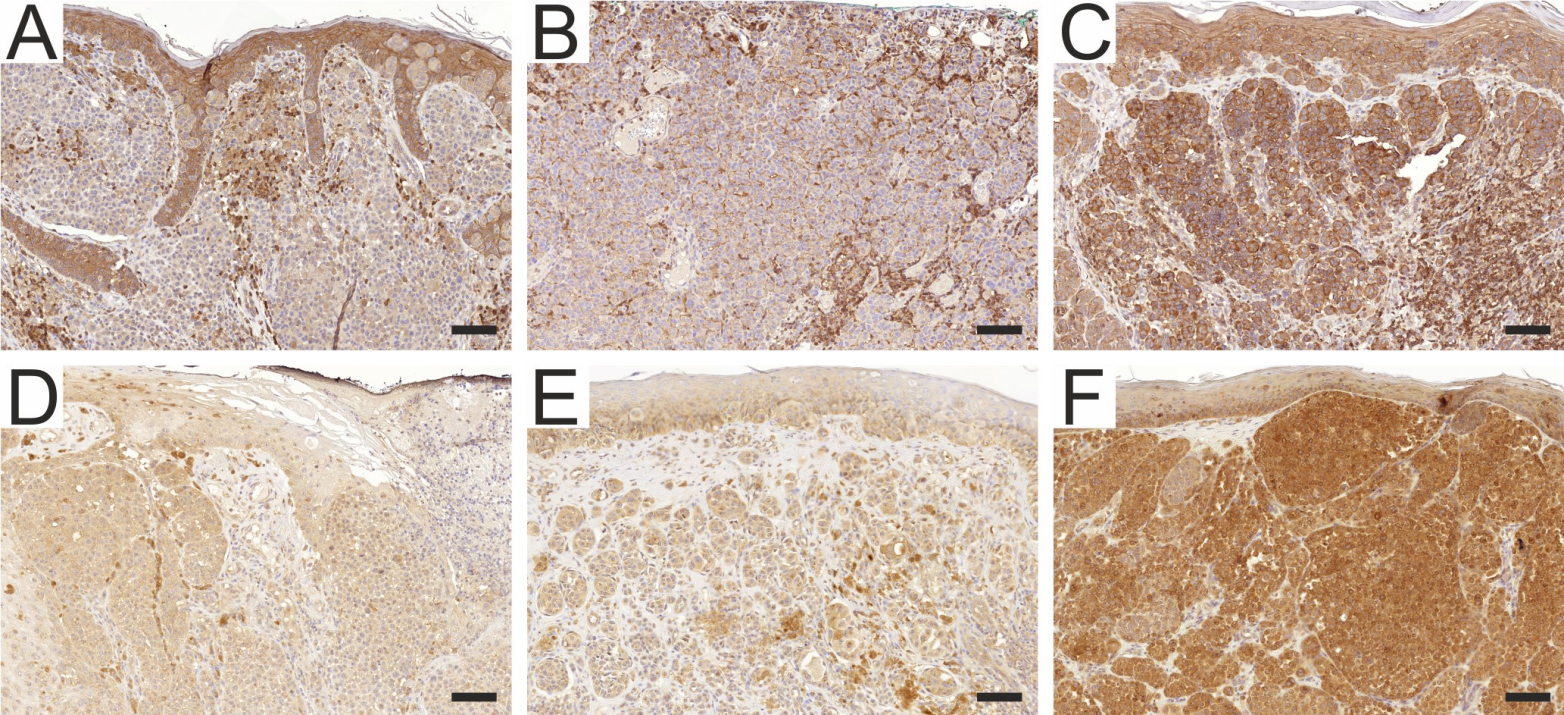
Examples of Ezrin and FMNL2 immunohistochemistry results in primary cutaneous melanomas. A = weak cytoplasmic ezrin reactivity. B = Moderate ezrin reactivity. C = Strong ezrin reactivity. D = Weak cytoplasmic FMNL2 reactivity. E = Moderate FMNL2 reactivity. F = Strong FMNL2 reactivity. Scalebar = 100μm.

For ezrin analysis, sections were reacted with the primary murine IgG mAb to human ezrin (clone 3C12, 1:250 dilution) [21], after deparaffinization and antigen retrieval with high pH Target retrieval solution (Dako, Glostrup, DK). Endogenous peroxidase activity was blocked with 3% hydrogen peroxidase, and nonspecific binding was prevented by using Novolink Protein block reagent. Immunoperoxidase staining was performed using a polymerized reporter enzyme staining system (Novolink Polymer Detection System, Leica Biosystems, Newcastle, GB) and ultraView Universal DAB Detection Kit (Ventana, Tucson, AZ) to visualize the bound antibody. In control experiments, antibody diluent replaced the primary antibody. The sections were counterstained with hematoxylin (Merck, Darmstadt, Germany) prior to mounting. The samples were evaluated by two blinded examiners (AM and JJ) and in case of discrepancy, a consensus was formed. Previously reported grading protocol was used where the expression levels are graded as negative, weak, moderate or strong [22]. Examples of different staining intensities are shown in Fig 1.

For FMNL2 analysis, specimens were stained with a rabbit anti-human FMNL2 polyclonal antibody (Sigma-Aldrich Corporation, St Louis, MO) (1:500). The antibody validation and staining procedures have been described previously [14]. We used the described intensity evaluation, where the basal layer of skin keratinocytes served as an internal reference. Staining intensity was divided into three categories: weak, moderate (similar to the basal keratinocytes) and strong. Staining intensity was evaluated by two investigators (AM and MG) blinded for the follow-up data and the sentinel node positivity. Examples of staining intensities are shown in Fig 1.

### Statistical analysis

Categorical variables were characterized using frequencies and percent and in case of continuous variables means, range of values was used. Cox's regression analysis and chi-square tests were used to determine the significant prognostic factors of disease free survival and recurrence free survival. Recurrence free survival was calculated from the date of the operation to the date of first local, regional or distant recurrence or to the end of the follow up. Significant explanatory variables in univariate analysis were included in the multivariate analysis. We also included parameters that were not significant in our material but are known to be important prognosticators in melanoma (*i*.*e*. ulceration and Breslow thickness). Cumulative percentages for survival were estimated using Kaplan-Meier technique and differences between the staining groups were tested using log-rank test. The results of Cox's regression analyses were quantified by calculating hazard ratios with 95% confidence intervals (95% CI). P-values ≤ 0.05 were regarded as statistically significant. All analyses were performed with SPSS-statistical program (IBM SPSS Statistics 24). All features of the individual tumors are presented in Supporting information.

## Results

The median age was 57.9 y (range 31–83 y). Of the 140 patients 72 were female and 68 were male. Location of the melanoma was most often trunk area (N = 64), followed by lower limb (N = 36), upper limb (N = 20) and head and neck (N = 20). The most common melanoma subtype was superficially spreading melanoma (N = 71), followed by nodular (N = 50) and acral (N = 6). Thirteen cases could not be categorized. In the entire cohort, ulceration was present in 32 (22.9%) primary melanomas (Table 1). We performed further division of the cohort in to smaller subgroups according to melanoma location and pathological characteristics. The only subgroup that remained sufficiently large for statistical analyses was the superficially spreading melanoma group (N = 71).

**Table 1:** Clinical and histopathological parameters of the patient cohort.

Age (years)		Cohort	SNB+	SNB-	p-value	HR (95%CI)
	Mean	57.9				
	Median	57				
	Range	31-83	Matched	Matched		
Follow-up (years)						
	Mean	5.1				
	Median	5.0				
	Range	0.2-10	Matched	Matched		
Gender		N (%)				
	Female	70 (50.0)				
	Male	70 (50.0)	Matched	Matched		
Location						
	Trunk	64 (45.7)				
	Lower Limb	36 (25.7)				
	Upper Limb	20 (14.3)				
	Head and neck	20 (14.3)	Matched	Matched		
Breslow thickness						
	≤ 1mm	17 (12.1)				
	1.01-2.0 mm	65 (46.4)				
	2.01-4.0 mm	58 (41.4)	Matched	Matched	NS	
Ulceration						
	Absent	108 (77.1)	54 (77.1)	54 (77.1)	NS	
	Present	32 (22.9)	16 (22.9)	16 (22.9)	NS	
Dermal mitoses						
	<1/mm2	33 (23.6)	12 (17.1)	21 (30.0)	NS	
	≥1/mm2	107 (76.4)	58 (82.9)	49 (70.0)	0.015	1.91 (1.13-3.22)
Regression						
	Yes	7 (5.0)	3 (4.3)	4 (5.7)	NS	
	No	133 (95.0)	67 (95.7)	66 (94.3)	NS	
Outcome						
	Alive	102 (72.9)	45 (64,3)	57 (81.4)	0.013	2.31 (1.12-4.45)
	Died of melanoma	26 (18.6)	22 (31.4)	4 (5.7)	<0.0001	7.16 (2.46-20.82)
	Died of other disease	12 (8.5)	3 (4.3)	9 (12.9)	NS	
	Recurrence	35 (25.0)	30 (42.9)	5(7.1)	<0.0001	8.25 (3.19-21.31)

SNB+ = Sentinel node positive patients of the cohort. SNB- = Sentinel node negative patients of the cohort. NS = not significant. HR (95%CI) = Hazard ratio, 95% confidence interval.

We first analyzed, whether any of the clinicopathological parameters or tissue biomarkers associated with sentinel node involvement. Mitotic count (≥1/mm^2^ vs. <1/mm^2^) correlated with sentinel node positivity (p = 0.036), whereas ulceration or tumor regression was not significantly different between the SNB+ and SNB- melanomas (Table 1). Neither was there a difference in FMNL2 or ezrin expression between SNB+ and SNB- tumors (Table 2). On the other hand, positive BRAF V600E immunoreactivity correlated with sentinel node involvement (p = 0.013) in the entire cohort. When subjected to multivariate analysis with clinically known prognostic markers of melanoma (Breslow thickness, ulceration, dermal mitosis level), BRAF remained as a significant independent prognostic factor for SNB positivity. (Table 2). Similar result was also seen in the subgroup of superficially spreading melanomas (p = 0.025).

**Table 2:** BRAF, FMNL2 and Ezrin staining intensities of the whole cohort.

N	Whole cohort	SNB +	SNB -	p-value	HR (95%CI)
	140	70	70		
BRAF-status					
+	59(42.1%)	37 (52.9%)	22 (31.4%)	0.013	1.82 (1.14-2.92)
-	81(57.9%)	33 (47.1%)	48 (68.6%)	0.013	
FMNL-2 status					
0 (negative)	-	-	-		
1 (weak)	14(10%)	7 (10%)	7 (10%)	NS	
2 (intermediate)	66(47.1%)	29 (41.4%)	37 (52.9%)	NS	
3 (Strong)	60(42.9%)	34 (48.6%)	26 (37.1%)	NS	
Ezrin status					
0 (negative)	5(3.6%)	-	5 (7.1%)	NS	
1 (weak)	67(47.9%)	39 (55.7%)	28 (40.0%)	NS	
2 (intermediate)	56(40%)	25 (35.7%)	31 (44.3%)	NS	
3 (Strong)	12(8.5%)	6 (8.6%)	6 (8.6%)	NS	

Note that positive BRAF V600E immunoreactivity correlated with sentinel node involvement (p = 0.010). SNB+ = Sentinel node positive patients of the cohort. SNB- = Sentinel node negative patients of the cohort, NS = not significant, HR(95%CI) = Hazard ratio, 95% confidence interval.

Twenty-six patients died of melanoma (22 in the SNB+ group and 4 in the SNB- group). In the whole cohort, overall survival was 72.9% and melanoma-specific survival 81.4%. As expected, SNB+-group showed decreased overall survival (OS, 64.3% vs. 81.4%) and melanoma-specific survival (MSS 68.6% vs. 94.3%). Recurrence rate was 30 (42.9%) in the SNB+ group and 5 (7.1%) in the SNB- group. Recurrence free survival (RFS) was 3.96 years in the SNB+ and 5.63 years in the SNB- group. Average time for a recurrence was 2.0 years. The outcome information of the patient cohort is summarized in Table 1.

**Fig 2:**
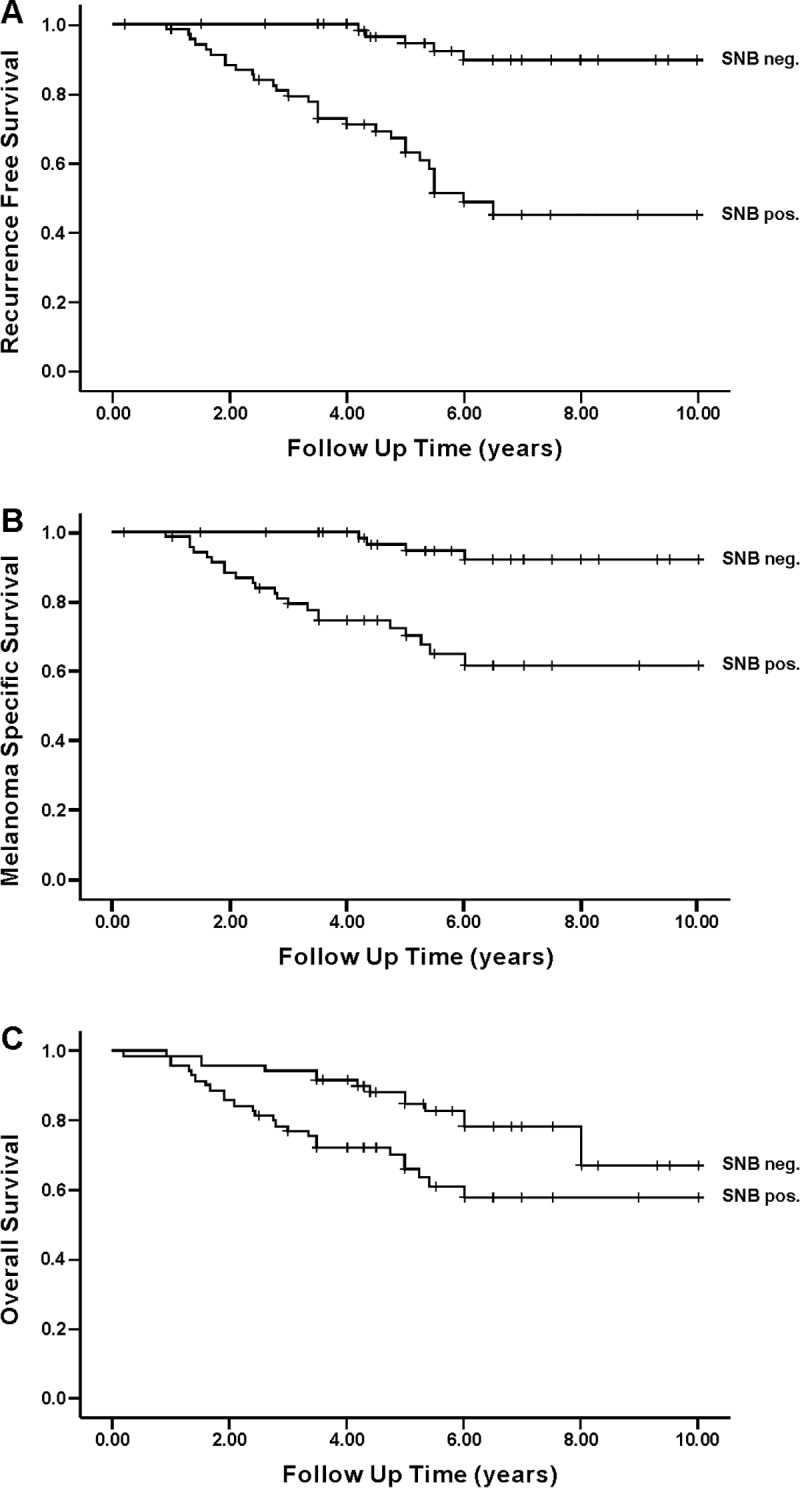
Kaplan-Meier analysis of intermediate thickness melanomas for recurrence free survival (RFS), melanoma specific survival (MSS) and overall survival (OS) in the whole cohort. (A) = RFS (p<0.0001). (B) = MSS (p<0.0001). (C) = OS (p = 0.025).

Sentinel node positivity was a significant prognostic factor for RFS (p<0.0001), MSS (p<0.0001) and OS (p = 0.013) in univariate analysis. Kaplan-Meier survival curves are shown in Fig 2. In this cohort of Breslow 0.9–4.0 mm melanomas, thickness of the primary tumor was not associated with RFS, MSS or OS. Neither did ulceration of the primary tumor correlate with RFS (p = 0.089), MSS (p = 0.127) or OS (p = 0.375), possibly due to the relatively small number of ulcerated tumors (N = 32).

In the superficially spreading melanoma subgroup, there were no significant differences between SNB+ and SNB- melanomas regarding ulceration, mitotic count and Breslow thickness. However, there was a strong correlation between sentinel node positivity and decreased OS (p = 0.005), MS (0.004) and RFS (p<0.0001). In this subgroup, high mitotic count correlated with OS (p = 0.047), MSS (p = 0.019) and RFS (p = 0.020). Clinical and histopathological parameters of this subgroup are shown in Table 3.

**Table 3:** Clinical and histopathological parameters of the Superficial Spreading Melanoma—subgroup.

N		SSM - group	SNB +	SNB-	p-value	HR (95%CI)
		71	33	38		
Age (years)						
	Mean	57.9	57	57		
Follow-up (years)						
	Mean	5.1	5.3	5.3		
Gender		N (%)				
	Female	32 (45.1)	14 (42.4)	18 (47.4)		
	Male	39 (54.9)	19 (57.6)	20 (52.6		
Location						
	Trunk	33 (46.5)	16 (48.5)	10 (26.3)		
	Lower Limb	20 (28.2)	7 (21.1)	17 (44.7)		
	Upper Limb	11 (15.5)	5 (15.2)	8 (21.1)		
	Head and neck	7 (9.8)	5 (15.2)	3 (7.9)		
Breslow thickness						
	≤ 1mm	11 (15.5)	4 (12.1)	7 (18.5)	NS	
	1.01-2.0 mm	34 (47.9)	18 (54.6)	20 (52.6)	NS	
	2.01-4.0 mm	26 (36.6)	11 (33.3)	11 (28.9)	NS	
Ulceration						
	Absent	58 (81.7)	25 (75.8)	33 (86.8)	NS	
	Present	13 (18.3)	8 (24.2)	5 (13.2)	NS	
Dermal mitoses						
	<1/mm2	18 (25.4)	6 (18.2)	14 (36.8)	NS	
	≥1/mm2	53 (74.6)	27 (81.8)	24 (63.2)	NS	
Outcome						
	Alive	54 (76.1)	21 (63.6)	35 (92.1)	0.005	6.30 (1.77-22.47)
	Died of melanoma	14 (19.7)	12 (36.4)	2 (5.3)	0.004	9.25 (2.06-41.57)
	Died of other disease	3 (4.2)	0	1 (2.6)	NS	
	Recurrence	20 (28,2)	17(51,5)	3 (12.7)	<0.0001	9.44 (2.76-32.35)

SSM-group = superficial spreading melanoma-group. SNB+ = Sentinel node positive patients of the subgroup. SNB- = Sentinel node negative patients of the subgroup. NS = not significant. HR (95%CI) = Hazard ratio, 95% confidence interval.

We further subjected sentinel node positivity to multivariate analysis of RFS, MSS and OS, together with dichotomized Breslow thickness (<1.95mm or ≥2.0mm), presence of ulceration and number of mitoses. SNB status remained as a significant independent prognostic factor for RFS (p<0.0001), MSS (p<0.004) and OS (p = 0.003).

While BRAF V600E immunoreactivity correlated with sentinel node positivity, we found no correlation with RFS, MSS or OS in the entire cohort. However, when evaluated in the superficially spreading melanoma subgroup, BRAF V600E positivity indicated adverse RFS (p = 0.039) and OS (p = 0.012). We found no association between the intensity of FMNL2 immunoreactivity and RFS, MSS or OS. Neither was FMNL2 intensity correlative in the superficially-spreading melanoma subgroup. Similarly, ezrin-staining intensity did not predict RFS, MSS or OS in the patient cohort (Table 2).

**Fig 3:**
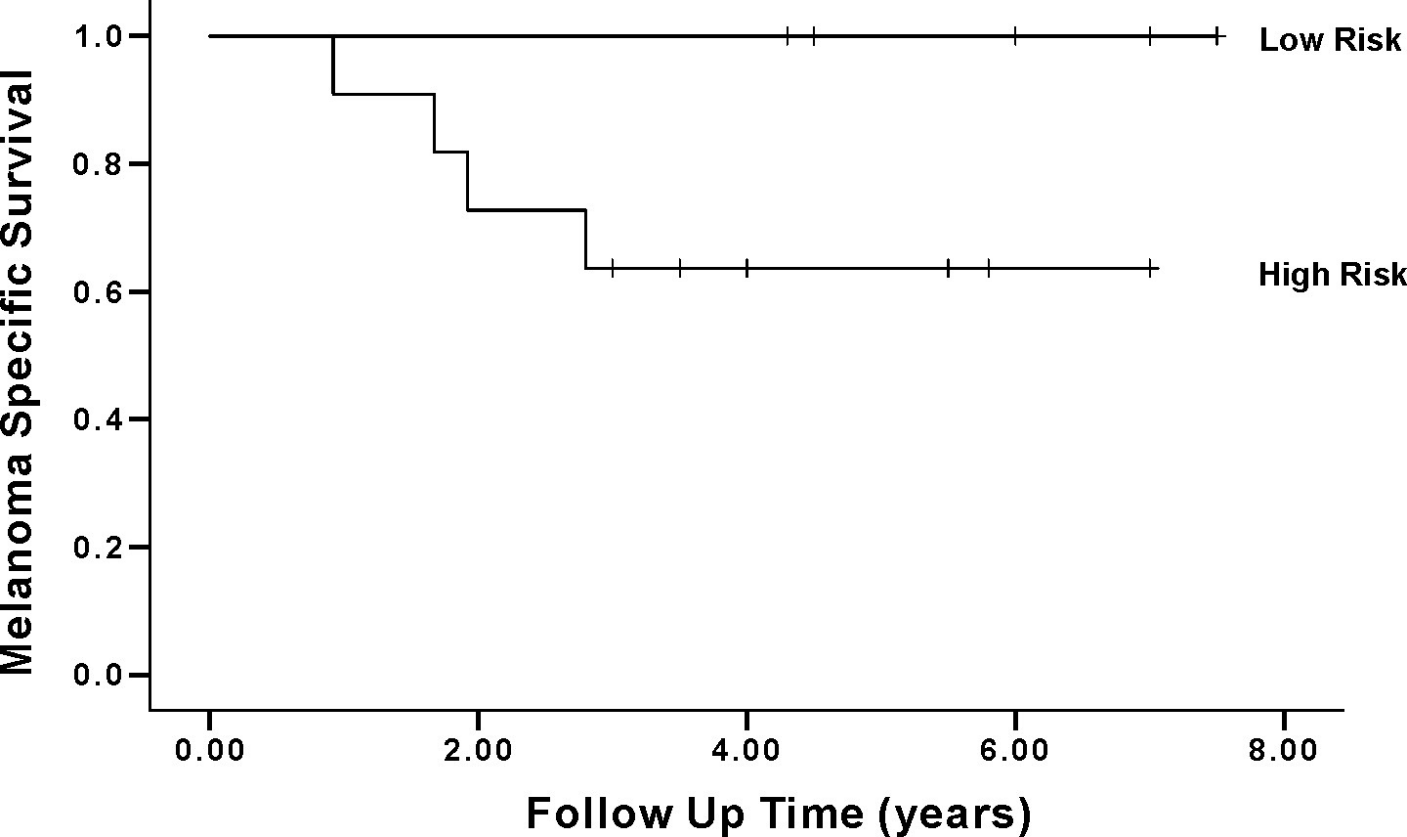
Kaplan-Meier survival analysis of intermediate thickness melanomas of high risk–and low risk–subgroups. High risk = Breslow ≥ 2mm, high mitotic count [≥ 1/mm2] + and BRAF V600E+, low risk = Breslow <2mm, low mitotic count, BRAF V600E-). Melanoma specific 5-year survival is significantly reduced in the High risk–group (p = 0.011).

Finally, we wanted to test, whether BRAF status (BRAF V600E IHC positive or BRAF V600E negative), in combination with clinical features (Breslow ≥ 2mm or <2mm and mitotic count ≥ 1/mm^2^ or <1/mm^2^) predicts SNB status and survival in the superficially spreading melanomas. Of the 71 patients, eleven qualified into a high risk group (all three features unfavorable) and eleven into a low-risk group (all three features favorable). In the high risk-group, SNB positivity was more common (72,7% vs. 18,2% p = 0.001) and recurrence significantly more frequent (54,5% vs. 0,0%, p = 0.003) than in low risk group. The 5-year melanoma-specific survival in the high-risk group was only 63,6%, which was significantly different from the low risk group, in which all patients were alive (p = 0.031). These two groups are shown in Table 4. Melanoma-specific survival functions are shown in Fig 3.

**Table 4:** “High risk” and “low risk”—groups.

	High risk (Breslow>2,High mitoses, BRAF+)	Low risk (Breslow<2, low mitoses,BRAF-)
**N**	11	11
**SNB+ (%)**	8 (72.7%)	2 (18.2%)
**Recurrence (%)**	6 (54.5%)	0 (0.0%)
**Melanoma survival**	7 (63.6%)	11 (100%)

SNB+ = Number of sentinel node positive patients in the two groups. Recurrence = Number of recurrent melanomas in the two groups.

## Discussion

Sentinel node biopsy provides important information for melanoma staging and for adjuvant treatment decisions. However, the current practice for selecting individuals for SNB is far from optimal, since only 20–25% of biopsied patients actually demonstrate lymph node involvement. In this study we wanted to test whether clinicopathological parameters or novel biomarkers, either alone or in combination, could indicate the probability of sentinel node involvement and thereby improve the selection of patients for SNB. In a matched case–control setting we demonstrate that mitotic count and BRAF V600E immunohistochemistry significantly correlate with SNB positivity in cutaneous melanomas of intermediate thickness. Our study also shows that in this matched cohort, SNB involvement is a strong independent predictor of RFS, MSS and OS. Finally, we show preliminary evidence that a combination of Breslow thickness, mitotic count and BRAF immunohistochemistry may identify a group within superficial spreading melanomas with an improved survival probability independent of SNB analysis.

To the best of our knowledge, this is the first report to show an association between BRAF V600E immunohistochemistry and sentinel lymph node involvement. Until now, BRAF analysis has typically been performed as part of clinical practice to find out, whether a patient would benefit from targeted inhibition of mutant BRAF. Some studies have also linked BRAF mutation with aggressive features [23]. In comparison with patients without BRAF mutations, BRAF-mutated melanomas are more likely to metastasize to the brain and may have a worse outcome. Our findings are in line with a recent study by Adler et al., in which BRAF mutation was associated with lymph node metastasis and sentinel lymph node positivity [24]. The major difference between our study and that of Adler et al. is the methodology for BRAF analysis. Here we used an immunohistochemical detection of mutant BRAF V600E instead of DNA sequencing used in the previous work. While the concordance between DNA sequencing and immunohistochemistry is excellent (20), both methods have their advantages. DNA analysis covers all BRAF variants, while the antibody is specific to BRAF V600E, and will not detect the rare additional oncogenic mutations. Especially elder patients with head and neck melanomas are prone to express BRAF V600K –mutation in their melanomas [25]. This subgroup of patients could thus prove to be a limitation for BRAF IHC usage in prognosticating melanomas. On the other hand, immunohistochemistry is faster, cheaper and readily available as part of routine pathology diagnostic arsenal. Furthermore, it can be performed on sparse specimens containing only small amounts of melanoma cells.

In our study, BRAF V600E mutation was associated with adverse RFS and OS in the superficially spreading melanomas, the histological subtype which typically harbors BRAF mutations. In most previous studies, a correlation between BRAF-mutation and shorter OS has been restricted to advanced metastatic disease, while the results in early stage melanomas have been conflicting (reviewed in [26]). There are, however, some earlier studies to support our finding on the predictive role for BRAF mutation also in early stage disease [27,28].

In our study FMNL2-expression was not an independent prognosticator of sentinel node involvement or melanoma outcome in contrast to previous results [15]. There may be several explanations for this apparent discrepancy. The cohorts in the two studies were significantly different, as the previous study included only lymph node negative cases, while in the current study half of the patients had sentinel node involvement. Moreover, here we excluded Breslow >4.0 mm melanomas, while in the previous study 20.1% of melanomas belonged to that group. Finally, the previous study had more ulcerated melanomas than the current cohort (43.2% vs. 18.3%). We conclude that further studies are needed to confirm the role of FMNL2 in melanoma biology. The fact that ezrin expression did not correlate with sentinel node positivity or outcome is not totally surprising, as most previous studies have linked ezrin expression to melanoma evolution, *e*.*g*. by demonstrating increased melanoma expression in metastases as compared with the primary tumor.

Not surprisingly, sentinel node status proved to be a significant prognostic factor in our material of intermediate thickness melanomas, both in the entire cohort and the superficially spreading subtype. This correlation and the role of SNB-status has been substantiated in numerous studies. Interestingly, independent of the sentinel node status, the combination of Breslow thickness, mitotic count and BRAF immunohistochemistry defined patient groups with highly different outcomes. A “low risk” group (Breslow <2mm, low mitotic count, BRAF V600E-), had a 100% 5-year survival, while the survival probability for the “high risk” group (Breslow >2mm, high mitotic count, BRAF V600E+, 18% of SSM) was just over 60%. It should be noted that while the statistical difference between the groups was significant, the group sizes were small. Therefore, further studies are needed to confirm, whether BRAF analysis in combination with conventional clinicopathological markers could provide meaningful outcome information for clinical decision support.

This study confirms the central role of sentinel node biopsy in melanoma prognostication, but the challenge of optimal stratification of patients for SNB remains. Our results demonstrate that BRAF immunohistochemistry could be an integral part in assisting selection of patients with intermediate thickness melanomas for sentinel node dissection. Further prospective studies are needed for validation of these findings.

## Supporting information

S1 Table Clinical features and staining results of individual tumors. (XLSX)

## References

[1] WHO International Agency of Cancer Research, Availabe from: http://globocan.iarc.fr/Pages/fact_sheets_population.aspx

[2] Guy GP Jr, Thomas CC, Thompson T, Watson M, Massetti GM, Richardson LC. Vital signs: melanoma incidence and mortality trends and projections—United States, 1982-2030.MMWR Morb Mortal Wkly Rep. 2015 6 5;64(21):591–6. 26042651 PMC4584771

[3] Gershenwald JE, Scolyer RA, Hess KR, Sondak VK, Long GV, Ross MI, et al Melanoma staging: Evidence-based changes in the American Joint Committee on Cancer eighth edition cancer staging manual. CA Cancer J Clin. 2017 11;67(6):472–492. 10.3322/caac.21409 29028110 PMC5978683

[4] Balch CM, Gershenwald JE, Soong SJ, Thompson JF, Atkins MB, Byrd DR, et al Final version of 2009 AJCC melanoma staging and classification. J Clin Oncol 2009; 27: 6199–6206. 10.1200/JCO.2009.23.4799 19917835 PMC2793035

[5] Chen J, Xu Y, Zhou Y, Wang Y, Zhu H, Shi Y. Prognostic role of sentinel lymph node biopsy for patients with cutaneous melanoma: A retrospective study of surveillance, epidemiology, and end-result population-based data. Oncotarget. 2016 7 19; 7(29): 45671–45677. 10.18632/oncotarget.10140 PMC5216751 27344178

[6] Wong SL, Brady MS, Busam KJ, Coit DG. Results of sentinel lymph node biopsy in patients with thin melanoma. Annals of Surgical Oncology 2006, Volume 13, Issue 3, pp 302–309. 10.1245/ASO.2006.02.021 16485151

[7] Morton DL, Thompson JF, Cochran AJ, Mozzillo N, Nieweg OE, Roses DF, et al Final trial report of sentinel-node biopsy versus nodal observation in melanoma. N Engl J Med 2014;370:599–609. 10.1056/NEJMoa1310460 24521106 PMC4058881

[8] Russo A, Ficili B, Candido S, Pezzino FM, Guarneri C, Biondi A, et al Emerging targeted therapies for melanoma treatment (review). Int J Oncol 2014; 45: 516–524. 10.3892/ijo.2014.2481 24899250 PMC4091965

[9] Libra M, Malaponte G, Navolanic PM, Gangemi P, Bevelacqua V, Proietti L, et al Analysis of BRAF mutation in primary and metastatic melanoma. Cell Cycle 2005; 4: 1382–1384. 10.4161/cc.4.10.2026 16096377

[10] Cheng L, Lopez-Beltran A, Massari F, MacLennan GT, Montironi R. Molecular testing for BRAF mutations to inform melanoma treatment decisions: a move toward precision medicine. Mod Pathol. 2018 1;31(1):24–38. 10.1038/modpathol.2017.104 29148538 PMC5758899

[11] Lu H, Liu S, Zhang G, Kwong LN, Zhu Y, Miller JP, et al Oncogenic BRAF-mediated Melanoma Cell Invasion. Cell Rep. 2016 5 31; 15(9): 2012–2024. 10.1016/j.celrep.2016.04.073 27210749 PMC4889462

[12] Faix J, Grosse R. Staying in shape with formins. Dev Cell. 2006 6;10(6):693–706. 10.1016/j.devcel.2006.05.001 16740473

[13] Block J, Breitsprecher D, Kühn S, Winterhoff M, Kage F, Geffers R, et al FMNL2 drives actin-based protrusion and migration downstream of Cdc42. Curr Biol. 2012 6 5;22(11):1005–12. 10.1016/j.cub.2012.03.064 22608513 PMC3765947

[14] Gardberg M, Talvinen K, Kaipio K, Iljin K, Kampf C, Uhlen M, et al Characterization of Diaphanous-related formin FMNL2 in human tissues. BMC Cell Biol. 2010 7 15;11:55 10.1186/1471-2121-11-55 20633255 PMC2912821

[15] Gardberg M, Heuser VD, Koskivuo I, Koivisto M, Carpén O. FMNL2/FMNL3 formins are linked with oncogenic pathways and predict melanoma outcome. J Pathol Clin Res. 2016 1 21;2(1):41–52. 10.1002/cjp2.34 27499915 PMC4858127

[16] Luo Y, Zheng C, Zhang J, Lu D, Zhuang J, Xing S, et al Recognition of CD146 as an ERM-binding protein offers novel mechanisms for melanoma cell migration. Oncogene 2012; 31: 306–321. 10.1038/onc.2011.244 21725352

[17] Curto M, McClatchey Al. Ezrin, a metastatic detERMinant? Cancer Cell 2004; 5: 113–114. 14998486 10.1016/s1535-6108(04)00031-5

[18] Mäkitie T, Carpén O, Vaheri A, Kivelä. Ezrin as a prognostic indicator and its relationship to tumor characteristics in uveal malignant melanoma. Invest Ophthalmol Vis Sci. 2001 10;42(11):2442–9. 11581181

[19] Zhu L, Ito T, Nakahara T, Nagae K, Fuyuno Y, Nakao M, et al Upregulation of S100P, receptor for advanced glycation products and ezrin in malignant melanoma. J Dermatol 2013 12; 40(12):973–9. 10.1111/1346-8138.12323 24303922

[20] Thiel A, Moza M, Kytölä S, Orpana A, Jahkola T, Hernberg M, et al Prospective immunohistochemical analysis of BRAF V600E mutation in melanoma. Hum Pathol. 2015 2;46(2):169–75. 10.1016/j.humpath.2014.08.018 25442222

[21] Böhling T, Turunen O, Jääskelainen J, Carpén O, Sainio M, Wahlström T, et al Ezrin expression in stromal cells of capillary hemangioblastoma. An immunohistochemical survey of brain tumors. Am J Pathol, 148 (1996), pp. 367–373. 8579099 PMC1861673

[22] Ilmonen S, Vaheri A, Asko-Seljavaara S, Carpén O. Ezrin in primary cutaneous melanoma. Mod Pathol. 2005 4;18(4):503–10. 10.1038/modpathol.3800300 15475929

[23] Long GV, Menzies AM, Nagrial AM, Haydu LE, Hamilton AL, Mann GJ, et al Prognostic and clinicopathologic associations of oncogenic BRAF in metastatic melanoma. J Clin Oncol 2011;29: 1239–1246. 10.1200/JCO.2010.32.4327 21343559

[24] Adler N, Wolfe R, Kelly JW, Haydon A, McArthur GA, McLean CA, et al Tumor mutation status and sites of metastasis in patients with cutaneous melanoma. Br J Cancer. 2017 9 26;117(7):1026–1035. 10.1038/bjc.2017.254 28787433 PMC5625668

[25] Menzies AM, Haydu LE, Visintin L, Carlino MS, Howle JR, Thompson JF, et al Distinguishing clinicopathologic features of patients with V600E and V600K BRAF-mutant metastatic melanoma. Clin Cancer Res. 2012 6 15;18(12):3242–9. 10.1158/1078-0432.CCR-12-0052 22535154

[26] Adler NR, Haydon A, McLean CA, Kelly JW, Mar VJ. Metastatic pathways in patients with cutaneous melanoma. Pigment Cell Melanoma Res. 2017 1;30(1):13–27. 10.1111/pcmr.12544 27900851

[27] Mar VJ, Liu W, Devitt B, Wong SQ, Dobrovic A, McArthur GA, et al The Role of BRAF mutations in primary melanoma growth rate and survival. Br. J. Dermatol. 2015 7;173(1):76–82. 10.1111/bjd.13756 25752325

[28] Nagore E, Requena C, Traves V, Guillen C, Hayward NK, Whiteman DC, Hacker E. Prognostic value of BRAF mutations in localized cutaneous melanoma. J. Am. Acad. Dermatol. 2014 5; 70(5):858–862. 10.1016/j.jaad.2013.10.064 24388723

